# Birth weight in a large series of triplets

**DOI:** 10.1186/1471-2431-11-24

**Published:** 2011-04-01

**Authors:** Diane J Lamb, Christel M Middeldorp, Catharina EM van Beijsterveldt, Jacqueline M Vink, Monique C Haak, Dorret I Boomsma

**Affiliations:** 1 Department of Biological Psychology, VU University, The Netherlands; 2 Department of Child and Adolescent Psychiatry, Academic Medical Center, The Netherlands; 3 Department of Child and Adolescent Psychiatry, GGZ inGeest/VU medical center, The Netherlands; 4 Department of Obstetrics and Gynecology, VU University Medical Center, The Netherlands

## Abstract

**Background:**

Triplets are often born premature and with a low birth weight. Because the incidence of triplet births is rare, there are relatively few studies describing triplet birth weight characteristics. Earlier studies are often characterized by small sample sizes and lack information on important background variables such as zygosity. The objective of this study is to examine factors associated with birth weight in a large, population-based sample of triplets registered with the Netherlands Twin Register (NTR).

**Methods:**

In a sample of 1230 triplets from 410 families, the effects of assisted reproductive techniques, zygosity, birth order, gestational age, sex, maternal smoking and alcohol consumption during pregnancy on birth weight were assessed. The resemblance among triplets for birth weight was estimated as a function of zygosity. Birth weight discordance within families was studied by the pair-wise difference between triplets, expressed as a percentage of the birth weight of the heaviest child. We compare data from triplets registered with the NTR with data from population records, which include live births, stillbirths and children that have deceased within days after birth.

**Results:**

There was no effect of assisted reproductive techniques on triplet birth weight. At gestational age 24 to 40 weeks triplets gained on average 130 grams per week; boys weighed 110 grams more than girls and triplets of smoking mothers weighted 104 grams less than children of non-smoking mothers. Monozygotic triplets had lower birth weights than di- and trizygotic triplets and birth weight discordance was smaller in monozygotic triplets than in di- and trizygotic triplets. The correlation in birth weight among monozygotic and dizygotic triplets was 0.42 and 0.32, respectively. In nearly two-thirds of the families, the heaviest and the lightest triplet had a birth weight discordance over 15%. The NTR sample is representative for the Dutch triplet population that is still alive 28 days after birth.

**Conclusion:**

Birth weight is an important determinant of childhood development. Triplet status, gestational age, sex, zygosity and maternal smoking affect birth weight. The combined effects amount to a difference of 364 grams between monozygotic girl triplets of smoking mothers compared to dizygotic boy triplets of non-smoking mothers of the same gestational age. Birth weight in triplets is also influenced by genetic factors, as indicated by a larger correlation in monozygotic than in di- and trizygotic triplets.

## Background

The incidence of triplet births is rare. In the Netherlands, up to 1980, a triplet birth occurred once per 10 thousand births. The number of triplet births increased after the introduction of assisted reproductive technologies (ART). In 1990, the number of triplet births had increased up to 6 per 10 thousand. From 2000 onwards, triplet birth rates decline again, mainly because of a change in policy of fertility clinics. The Central Bureau of Statistics of the Netherlands has monitored triplet birth rates at 2 per 10 thousand births since 2005 [1].

The prevalence of low birth weight (BW) and preterm deliveries is high in triplet births. Both low BW and prematurity are risk factors for adverse health, cognitive and behavioral outcomes later in life, e.g. see Arnoudse-Moens et al. (2009) and Bhutta et al. (2002) [2,3]. Among the factors that influence birth weight gestational age (GA) is the most important factor [4-6]. Alexander et al. [7]described how fetal growth in triplets does not follow the growth curves of singletons or twins. Triplet growth is characterized by different phases. In phase A, up to 26 weeks, triplet fetal growth is comparable to that of singletons. Phase B is roughly between 26 and 30 weeks. During phase B, there is a steady decrease in triplet growth relative to singletons, up to a difference of 20%. This is hypothesized to be due to the restricted intrauterine space. During phase C, 30 to 35 weeks, there is no further decrease relative to singletons. Triplet weight during that period is about 20% less than that of singletons. These three phases are also seen in twins, though later in time and to a lesser extent. Phase D is only seen in triplets and starts from a GA of about 35 weeks. In this phase, a marked decrease in triplet weight compared to that of singletons is seen. However only 10 - 13% of the triplets reach a GA of more than 35 weeks [4,8].

Other factors involved in triplet BW include sex, zygosity and birth order. As in singletons, boy triplets weigh more than girls [9,10]. In twins, dizygotic (DZ) twins weigh more than monozygotic (MZ) twins. This is mainly an effect of sharing a placenta. MZ twins are, compared to DZ twins, more in competition for nutrients [11,12]. In triplets a similar effect is found [8,13]. However, until now the effect of zygosity on BW in triplets has been based on small samples, and a distinction within DZ trios between the MZ pair and the DZ triplet has not always been made. Only a few studies specifically focused on birth order in twin and triplet pregnancies. These studies suggest that the first-born child is often the heaviest, followed by the second born child. In triplets, the third born child most often weighs the least [6,14].

Not all three children in triplet pregnancies are similarly affected with regard to BW. Inter-triplet BW discordance is thought to be a direct effect of physiological adaption to the limited uterine environment. One triplet grows at the expense of his brother or sister. Compared to twins, BW discordance in triplets is less well documented, although the phenomenon seems to be more common in triplets than in twins [15]. Especially severe discordance - defined as a difference in BW of over 35% - is higher in triplets than in twins: 9.5% in triplets compared to 3.1% in twins [16,17].

Maternal smoking during pregnancy is a known predictor for low BW in children [18,19]. A study in twins found a negative effect of maternal smoking on the regression of BW on gestational age. Hence, the twins of non-smoking mothers had a more optimal development of BW [20]. The effect of maternal alcohol consumption during pregnancy is less clear. Some studies in singletons suggested that alcohol consumption is unrelated to BW when corrected for GA [21,22]. Other studies in singletons showed an effect in mothers who consume more than 100 grams or more than 5 drinks per week [18,23], as well as an interaction between alcohol consumption and smoking during pregnancy. The effect of maternal smoking combined with maternal alcohol consumption on children's BW is larger than the summed effect of each separate causal agent [18,19,24]. As triplets are already more growth restricted compared to twins, the effects of maternal smoking and alcohol consumption could be even more detrimental. To our knowledge, no other studies have directly looked at the effect of maternal smoking and alcohol consumption during pregnancy on triplet BW.

In the past two decades, around 37% of the triplets born in the Netherlands have been registered with the Netherlands Twin Register (NTR). In this study we present descriptive statistics on triplet BW and analyses of the effect of sex, zygosity, birth order, GA, and maternal smoking and alcohol consumption during pregnancy. Correlations in triplet BW are calculated as a function of zygosity to investigate the role of genetic factors on BW. Lastly, BW discordance is described. We compare characteristics of triplets registered with the NTR with data from the Netherlands Perinatal Registry (NPR, [25]). Data from the NPR consists of the total group of triplets born in the Netherlands, including the stillbirths and children that decease soon after birth.

## Method

### Subjects

We use the term 'triplet' to denote one of three individuals born at the same birth, and refer to a 'trio' as three triplets born at the same birth. In total, 1966 triplets from 664 families were registered with the NTR. The sample includes 642 complete trios and 22 incomplete trios. The complete trios consisted of 125 trios comprising 3 females, 187 trios consisting of 1 male and 2 females, 207 trios consisting of 2 males en 1 female, and 123 trios comprising 3 males. The incomplete trios consisted of 18 males and 22 females. Trios were incomplete for various reasons (e.g. in young triplets: one of the triplets was deceased; in adult triplets: not all members of a trio participated).

**Figure 1:**
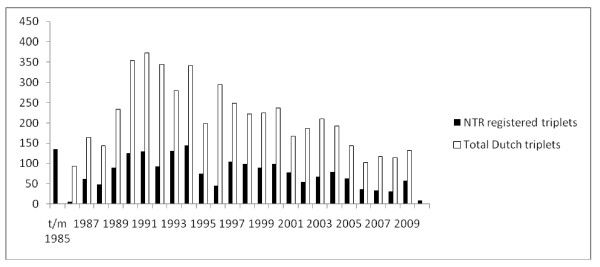
**Number of triplets born in the Netherlands and number of triplets registered at the Netherlands Twin Register (NTR) as a function of birth cohort**.

The Adult NTR (ANTR) registers multiples who are recruited as adults and the Young NTR (YNTR) registers multiples at birth. In figure 1 the number of triplets per birth cohort is given. Note that birth cohort 1986 marks the division between the ANTR and YNTR, triplets born after 1986 are registered with the YNTR. The oldest trio registered with the NTR were born in 1939. Data on triplet BW came from triplets born between 1970 and 2006.

To investigate the representativeness of the NTR triplet sample, we compared our sample with the Dutch triplet population regarding parity, BW, GA and age of the triplet mother when giving birth. In addition, we investigated factors that could have influenced non-response by comparing the study sample with trios who were registered with the NTR but did not participate in our surveys. The responders and non-responders were compared on age of the mother when giving birth, maternal education and population density.

### Data collection

Table 1 gives a summary of data collected in triplets. Three questionnaires include items regarding pregnancy, delivery and BW. Questionnaire 1 (Q1) is completed by mothers of YNTR triplets just after registration. Q1 inquired about the pregnancy (ART, gestational age, smoking and alcohol consumption during pregnancy and mode of delivery) and characteristics of the triplet (birth date, sex, domicile and birth weight) and about characteristics of the parents. Over the years, Q1 has been collected in 323 trios. In 2008, Questionnaire 2 (Q2) was sent to mothers of all triplets born before 2006. In addition to the questions in Q1, Q2 inquired about the characteristics of the triplets up to age 2 (e.g. growth curves, health and temperament). Q2 was sent out to 535 mothers and was returned by 264 mothers. Since the data collection in 2008, all mothers of triplets who reach age 2 receive Q2.

**Table 1:** Data collection in NTR triplets

Questionnaire	N trio list retour	N triplet list retour	Response rate
Q familial twinning	269		80%
Q1	323		58%
Q2	264		49%
CBCL age 3 - 12 mother		516	54 - 68%
CBCL age 3 - 12 father		510	54 - 68%
TRF consent form age 6 - 12	106		62%
TRF age 6 - 12		188	78%
YSR age 14 - 18		212	30 - 48%
ANTR surveys (list 1 to 8; ongoing)		315	47 - 48%

CBCL = Child Behavior Check List; TRF = Teacher Report Form; YSR = Youth Self Report; NTR = Netherlands Twin Register; ANTR = Adult NTR. Trio list are surveys inquiring about the trio, triplet list are surveys inquiring about a triplet.

In 2005, a questionnaire on familial twinning (Qft) was sent to all ANTR and YNTR mothers of multiples in the NTR [26,27]. This survey inquired about the occurrence of multiple births within a family, mode of conception, information on delivery and parental characteristics.

The Child Behavior Check List (CBCL; [28]) was sent multiple times to parents of triplets between age 3 and age 12 of the children. In addition, a short general questionnaire on parental and triplet characteristics (e.g. parental employment and religion, triplet school achievements and health) was included.

The Youth Self Report (YSR; [29,30]) was sent to YNTR triplets aged 14, 16 and 18. Triplets born between 1987 and 1992 were sent a paper and pencil version. Birth cohorts 1994 - 1995 received the YSR through the internet at ages 14 or 15. Birth cohort 1992 - 1993 completed the YSR via internet at age 16.

Starting in 2009, the Teacher Report Form (TRF; [29]) is collected. In 2009 we asked parents of 170 trios for consent to approach the children's teachers. 106 parents returned the consent form and 80 gave permission. After the parental consent was obtained, teachers of 240 triplets aged 6 to 12 received the TRF.

Triplets who agreed to participate in ANTR research have usually been included in the ANTR data collection. The procedure of data collection of the ANTR is described elsewhere [31,32].

For the current study, we used the data on BW, smoking and alcohol consumption during pregnancy acquired from questionnaire Q1 and Q2, completed by the mother of the triplets. Data on ART came from Q1, Q2 or Qft. Data on BW and zygosity were available for 455 and 465 trios, respectively, out of the 642 complete trios. For 433 trios information on both was available. For 410 trios, data were available for all variables under study, i.e. BW, zygosity, GA, alcohol consumption and smoking. Information on ART was available for 329 out of these 410 trios.

Response consistency was investigated by correlating responses given at subsequent questionnaires. When comparing Q1 and Q2, correlation was 0.93 (N = 521) for BW, 0.94 (N = 182) for GA, 0.95 (N = 182) for smoking, 0.57 (N = 179) for alcohol consumption and 0.98 (N = 100) for ART. Correlation for ART between Q1 and Qft was 0.95 (N = 132), and for ART between Q2 and Qft it was 1.00 (N = 132). Except for alcohol consumption, responses were highly consistent. For alcohol consumption, 14 mothers reported to have consumed alcohol on Q1 but not on Q2, and 7 mothers reported to have consumed alcohol on Q2, but not on Q1. In these cases, the response given at Q1 was used in the analysis.

### Statistical analyses

Analyses were performed using the software package Mx, which allows modeling of the dependency that exists between measures of pairs of relatives [33]. We tested the effect of zygosity, birth order, sex, GA, smoking and alcohol consumption on mean BW, and the effect of zygosity on variance and covariance in triplet BW. The effect of ART was tested in a smaller sub set of triplets in which information on ART was available.

First a full model, in which all effects were estimated, was fitted to the data. Subsequently, nested sub models were tested. In the full model the following parameters were estimated: the grand mean BW as a function of birth order and zygosity, the variance and covariance in BW (as a function of zygosity), and the regression coefficients on BW of GA, GA^2^, sex, alcohol consumption and smoking. In step 2, the means, variances and covariances of the MZ triplets from the MZ group were tested for equality with the MZ triplets from the DZ trio group. In step 3, the means, variances and covariances of the single remaining triplets in a DZ trio, not part of the MZ pair, and TZ triplets were constrained to be equal. In step 4, birth order effects on mean BW were tested within the zygosity groups. Finally, in step 5 it was tested whether the effects of GA, GA^2^, sex, maternal smoking and alcohol consumption on mean BW significantly differed from zero.

Using the raw likelihood method as implemented in Mx, the different models were compared using the log-likelihood ratio test. The difference in -2 times the log-likelihood (-2LL) between two nested models has a *χ² *distribution with the degrees of freedom (*df*) equaling the difference in *df *between the two models. A p-value of 0.05 was used to determine statistical significance.

In the regression analysis, sex was coded 0 for boys and 1 for girls and GA was coded as actual GA minus 40, ranging from 0 (GA of 40 weeks) to -16 (GA of 24 weeks). A possible flattening of triplet BW at the highest GA's was modeled with GA^2^. GA^2 ^was calculated by squaring the normalized score of the variable GA as described above (*Mean *= 0, *SD *= 1). Smoking and alcohol consumption during pregnancy were analyzed as dichotomous traits: yes (1) or no (0).

The MZ and DZ correlations were used to infer the influence of genetic and/or environmental factors on triplet BW. MZ triplet pairs and trios are genetically identical, whereas DZ and TZ triplet pairs and trios share on average 50% of their genetic material. A MZ correlation that is higher than the DZ correlation implies the influence of genetic factors. A DZ correlation that is higher than half the MZ correlation implies that shared environmental factors influence BW.

### Birth weight discordance

Three definitions are commonly used for BW discordance in twins. First, an absolute definition: the absolute difference in BW. Second, a percentage definition: BW disparity is calculated as a percentage of the largest child's BW. Third, a statistical definition: BW as percentile of one or two standard deviations from the mean [34]. Studies on trios often adopt the percentage definition. This means that the difference between the BW of the lightest and the heaviest triplet is calculated as an percentage of the BW of the heaviest [35].

The third method, specific to trios, takes into account the BW of the triplet that falls in between the heaviest and lightest triplets. In this method, a relative BW ratio is calculated by taking the difference between the middle and lightest triplet as a percentage of the difference between the heaviest and lightest triplet [16]. An advantage of this method is that this ratio is representative for situations in which the trio consists of 2 heavy and 1 light triplet or trio of 1 heavy and 2 light weights.

We used the percentage definition to estimate birth weight discordance, as this definition is most frequently used.

## Results

### Representativeness of the NTR triplet sample

Table 2 shows parity, BW, GA and age of mother for 1) the total group of Dutch triplets born in the Netherlands between 2000 and 2006 (the NPR gathered information on birth characteristics starting from birth cohort 2000), 2) the total group of triplets but without the trios in which one or more children were deceased before 28 days after birth, 3) the NTR sample. As can be seen, the NTR sample is highly comparable to the second group, but less to the first group, which contains more primiparous mothers. In the first group, GA is shorter and BW is lower. This indicates that the NTR sample is representative of trios with a favorable outcome, i.e. children that are still alive 28 days after birth.

**Table 2:** Descriptive statistics for a complete group of triplets born in the Netherlands between 2000 and 2006, a subset from this group including all triplets still alive after 28 days, and the NTR sample

	Dutch triplets (cohort 2000 - 2006)		Dutch triplets (cohort 2000 - 2006) alive after 28 days		NTR sample	
Cohort	2000 - 2006		2000 - 2006		1970 - 2006	
Parity (% primiparous)	54.7		50.9		51.9	

	M (sd)	N	M (sd)	N	M (sd)	N

BW	1745 (688)	1324	1920 (546)	1092	1900 (471)	1219
GA	32.3 (4.1)	1323	33.4 (2.8)	1092	33.7 (2.6)	1219
Age mother	31.6 (4.3)	486	31.7 (4.2)	379	30.8 (3.6)	348

BW = birth weight; GA = gestational age; NTR = Netherlands Twin Register.

Comparing the current study sample with trios registered in the NTR but not participating in this survey yielded no significant age difference between the two groups (*t*(454) = 1.90, *p *= 0.06). There was a difference in maternal education (*χ*^2^(3) = 8.69, *p *= 0.03). Maternal education in the responders group was lower than the educational level of mothers from the non-responders group (percentages of low, middle, middle high and high education: 12.4%, 36.0%, 29.4% and 22.2% for the responders versus 4.2%, 41.5%, 30.5% and 23.7% for the non-responders). There was also a difference in population density between the two groups (*χ*^2^(3) = 8.16, *p *= 0.00). Population density was categorized as more than or less than 1000 persons per square meters. The distribution in the response group was 50.0% and 50.0% and in the non-response group 43.4% and 56.6%, for < 1000 and > 1000 persons per square meters, respectively.

### Zygosity

Trio zygosity was determined by DNA, blood group assessments [36], or survey questions. The survey questions pertained to resemblance in hair, eye, and face color, and facial appearance, of each triplet pair in a trio. Furthermore, items were included inquiring if the triplets were ever mistaken for each other by family members or strangers. When DNA, blood or survey questions were not available, self or parental report on zygosity was adopted. Self or parental report on zygosity was based on the answer to two survey questions "What do you think the zygosity of the trio is?" and "And if the trio is a DZ trio, which pair forms the MZ pair?".

DNA samples were available for 79 triplets from 31 families and blood samples for 65 triplets from 22 families. Both DNA and blood samples were available for 47 triplets from 16 families. Survey questions about resemblance and self or parental report on zygosity were available for 318 and 450 trios, respectively. Zygosity estimate was based on the trio. This signifies that if one pair wise comparison could be made but information on the third triplet was missing, trio zygosity could not be determined. There were 22 trios with zygosity based on DNA and/or blood information on all triplets. Seventeen trios had information on zygosity based on both DNA and survey questions regarding resemblance. This provided the opportunity to look at the reliability of the survey information. Pair wise comparisons were incorrect in 10% of the cases. This is comparable with zygosity determination based on survey questions on resemblance in twins [37,38]. However, zygosity determination in trios is based on three pair wise comparisons. DNA and survey questions on resemblance gave the same zygosity result for 12 of these 17 trios. For 5 trios, survey questions on resemblance suggested that the trio was dizygotic while DNA determined that the trios were trizygotic. In 4 of these last 5 cases, self and/or parental report also suggested that the trios were dizygotic. Pairs determined as MZ where checked on sex (an opposite-sex pair cannot be MZ). This resulted in a zygosity determination of 465 triplet trios as presented in Table 3.

**Table 3:** Triplet sex and zygosity

	FFF	MFF	MMF	MMM	Total zygosity
MZ	23	-	-	23	46 (10%)
DZ	32	31	32	28	123 (26%)
TZ	31	106	125	34	296 (64%)

Total sex:	86	137	157	85	465

MZ = monozygotic; DZ = dizygotic; TZ = trizygotic; FFF = trio of three females; MFF = trio of 1 male and 2 females; MMF = trio of 2 males and 1 female; MMM = trio of three males.

### ART

ART are more commonly seen in triplet pregnancies than in twin or singleton pregnancies. We asked the triplet mothers in 350 returned questionnaires about the possible use of ART. 127 answered that the pregnancy was spontaneous, 103 after in vitro fertilization, 17 after intracytoplasmic sperm injection, 25 after intrauterine insemination, and 63 after ovulation induction with hormone tablets or subcutaneous injections. The remaining 15 mothers gave an unclear or no answer to this question. The age of the triplet mothers who made use of ART ranged from 21 to 43 years (M = 31, SD = 3.5), the age of mothers who spontaneously conceived the trio ranged from 20 to 41 years (M = 30, SD = 3.8). ART was overrepresented in the TZ triplet group. 87% of the mothers of TZ triplets reported ART, compared to 19% of the mothers of DZ's and only 3% of the mothers of MZ triplets.

Within the subset of triplets with information on ART, we did not find a significant effect of ART on BW (*χ^2^_(1) _*= 0.23, *p *= 0.63). ART was also tested in the TZ triplet group alone to correct for a possible confounding effect of zygosity, as TZ triplets are possibly heavier and overrepresented in the group of triplets born after ART. Still, no effect was found (*χ^2^_(1) _*= 1.23, *p *= 0.27). All other analyses were therefore performed on the total set of triplets, including triplets without information on ART.

### Birth weight

Descriptive statistics of the observed data are given in Table 4. The total sample with complete data included 37 MZ trios, 102 DZ trios which consist of one MZ pair and one DZ triplet, and 271 TZ trios. Mean GA of the triplets was little above 33 weeks. 26% of the triplets was born after a caesarean section. Only 3% of the mothers of triplets both smoked and consumed alcohol, while 15% reported smoking and 11% reported consuming alcohol during the pregnancy.

**Table 4:** Descriptive statistics of the raw scores on triplet BW and GA, and percentage of smoking and alcohol consumption during pregnancy, as a function of zygosity

	MZ		DZ	TZ
N trios	37		102	271
N boys/girls	41/70		145/161	387/426
GA mean (sd)	33.3 (2.15)		33.6 (2.89)	33.7 (2.52)
N mothers consuming alcohol	3 (8%)		19 (19%)	36 (13%)
N mothers smoking	6 (16%)		19 (19%)	49 (18%)

	MZ	MZ*	DZ**	TZ

Mean BW first born (grams)	1852	1952	2017	1954
Mean BW second born (grams)	1759	1887	1941	1933
Mean BW third born (grams)	1789	1831	2107	1829
Variance triplet BW	157	230	262	215
Pair-wise covariance triplet BW	110	162	156	144
Pair-wise correlation triplet BW	0.70	0.70	0.64	0.67
SGA first born (% SE ss/US ss/US ts)	19/42/3	22/38/5	27/37/7	25/44/7
SGA sec. born (% SE ss/US ss/US ts )	41/57/14	41/24/5	14/59/13	25/41/9
SGA third born (% SE ss/US ss/US ts)	32/51/16	35/39/8	18/33/11	18/52/15

*MZ triplets from DZ trios; ** DZ triplets from DZ trios; GA = gestational age; BW = birth weight; SGA = small for gestational age; US ss = United States' singleton standard; US ts = US triplet standard; SE ss = Swedisch singleton standard; MZ = monozygotic; DZ = dizygotic; TZ = trizygotic.

The uncorrected data on BW are presented as a function of zygosity and birth order. For the DZ trios two columns are presented. One column gives mean BW for the MZ pairs within the trio. The other column gives mean BW for the single remaining DZ triplets that are not part of the MZ pair.

Table 4 also shows the percentage of triplets who are small for gestational age (SGA). The 10^th ^percentile of BW as a function of GA is often classified as SGA. However, this 10^th ^percentile differs between singletons and triplets. For example, Alexander et al. (1998) [7] reported that for a GA of 33 weeks the singleton 10^th ^percentile of BW is 1673 grams, but for triplets it is only 1418 grams. The discrepancy between singletons and triplets increases with increasing GA. As such data are not available for Dutch triplets, we present the percentages of triplets who are SGA based on singleton standards for the United States (US) and based on US triplet standards as reported by Alexander in addition to Swedish singleton standards which are comparable to Dutch singleton standards [39,40].

Fit statistics of all tested models are presented in Table 5. The effects of GA, sex and smoking on mean BW as well as the variance, covariance and correlations of BW within MZ and DZ triplets are shown in Table 6. We found no difference between the mean, variance and covariance of triplets from the MZ group and MZ triplets from the DZ group (step 2). There were no significant differences in the variances and covariances of the DZ and TZ triplets, but there were differences between the means of the DZ triplets and the TZ triplets (*χ^2^_(3) _*= 16.57, *p *= 0.00). A significant birth order effect was found within the TZ group (*χ^2^_(2) _*= 29.07, *p *= 0.00), but not in the MZ and DZ group (*χ^2^_(2) _*= 5.61, *p *= 0.06 and *χ^2^_(2) _*= 0.92, *p *= 0.63, respectively).

**Table 5:** Model fitting results of the means model on triplet BW

step	Model	N_parameters_	-2LL	df	to model	*Χ^2^*	Δ df	*p*
1	Full model	25	576.10	1194				

2	a. Equal mean MZ pairs within MZ and DZ trios	22	577.11	1197	1	1.01	3	0.80
	b. Equal var MZ pairs within MZ and DZ trios	21	579.62	1198	2a	2.51	1	0.11
	c. Equal cov MZ pairs within MZ and DZ trios	20	581.32	1199	2b	1.70	1	0.19

3	a. Equal mean DZ/TZ	17	597.89	1202	2c	16.57	3	'0.00
	b. Equal var DZ/TZ	19	584.16	1200	2c	2.84	1	0.09
	c. Equal cov DZ/TZ	18	585.71	1201	3b	1.01	2	0.31

4	a. Equal mean within MZ	16	591.32	1203	3c	5.61	2	0.06
	b. Equal mean within DZ	14	592.24	1205	4a	0.92	2	0.63
	c. Equal mean within TZ	12	621.31	1207	4b	29.07	2	0.00

5	a. Effect GA^2 ^= 0	13	594.73	1206	4b	2.49	1	0.11
	b. Effect GA = 0	12	1013.26	1207	5a	418.53	1	0.00
	c. Effect sex = 0	12	631.42	1207	5a	36.69	1	0.00
	**d. Effect alcohol = 0**	**12**	**594.84**	**1207**	**5a**	**0.11**	**1**	**0.74**
	e. Effect smoking = 0	11	605.74	1208	5d	10.9	1	0.00

MZ = monozygotic; DZ = dizygotic; TZ = trizygotic; GA = gestational age. The best fitting model is printed in bold font.

**Table 6:** Parameter estimates for triplet BW and causal agents influencing BW, based on model 5d

	Mean/variance/covariance (grams)	
	MZ	DZ/TZ _(fb, sb, tb)_
Mean	2765	2915/2826, 2811, 2710
Variance	111	104
covariance	46	33
correlation	0.42 (0.29 - 0.54)	0.32 (0.25 - 0.38)

covariate	range	Effect in grams

GA	-16 to 0: weight gain per week	130
Sex	0/1: Boy/girl	-110
Smoking	0/1: No/yes	-104

GA = gestational age, fb = first born, sb = second born, tb = third born. Results given for MZ triplets include triplets from both the MZ group and the DZ group.

The tests of the fixed effects showed that GA was the most important contributor to mean BW in triplets. Between a GA of 24 to 40 weeks, the triplets gained 130 grams per week. No significant flattening of the growth line (GA^2^) was observed (*χ^2^_(1) _*= 2.49, *p *= 0.11). An effect of sex was found with boys being 110 grams heavier than girls (*χ^2^_(1) _*= 36.69, *p *= 0.00). Furthermore, triplets from mothers who smoked during pregnancy were 104 grams lighter than the triplets of mothers who did not smoke (*χ^2^_(1) _*= 10.9, *p *= 0.00). We did not find a significant effect of alcohol consumption during the pregnancy on triplet BW (*χ^2^_(1) _*= 0.11, *p *= 0.74).

Correlations in triplet BW as a function of zygosity were calculated before and after including the effects of GA, sex and smoking on mean BW in the model. Before correction, the MZ correlation was 0.70, and the DZ and TZ correlations were 0.64 and 0.67 respectively. Correlations in triplet BW were lower when the effects of GA, sex and smoking were included, indicating that these variables explain part of the resemblance in triplet BW. Furthermore, the MZ triplet correlation is higher than the DZ triplet correlation, 0.42 compared to 0.32, respectively. This indicates that in addition to common environmental effects, genetic factors also explain part of the variance in BW.

**Figure 2:**
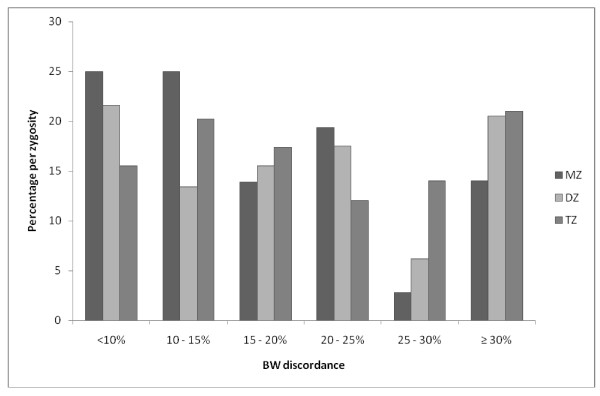
**Percentage triplets per BW discordance category as a function of zygosity**. BW = birth weight; MZ = monozygotic; DZ = dizygotic; TZ = trizygotic.

Finally, BW discordance was calculated. We compared the heaviest and the lightest triplet of a trio and found that in only 17.9% of the trios, BW discordance was less than 10%. In 60.6% of the trios BW discordance was between 10 - 30% and in 21.5% BW discordance was more than 30%. A total overview of the BW discordance distribution is given in table 7. Figure 2 presents BW discordance as a function of zygosity. There are more MZ triplets in the low discordance categories compared to the DZ and TZ triplets, and less MZ triplets compared to DZ and TZ triplets in the high discordance categories. This is in line with the higher correlation in BW in MZ triplets as reported above.

**Table 7:** Percentages of triplet pairs per BW discordance category

BW discordance	Heaviest - lightest	Heaviest - middle	Middle - ligthest
< 10%	17.9/14.8%	60.1/60.0%	48.6/47.7%
10 - 14.9%	18.9/19.1%	18.9/17.2%	18.7/19.1%
15 - 19.9%	16.6/19.6%	12.0/11.7%	12.8/11.2%
20 - 24.9%	14.1/12.8%	4.9/5.1%	5.6/5.4%
25 - 29.9%	11/7.9%	1.8/2.5%	4.9/4.9%
≥ 30%	21.5/25.8%	2.3/3.5%	9.5/11.7%

BW = birth weight. First presented percentage is calculated from triplets registered at the NTR, het second percentage is based on a national sample of triplets born in the Netherlands between 2000 and 2006, including triplets that deceased before 28 days after birth.

## Discussion

The present study describes the influence of genetic and environmental risk factors on BW in a large population based sample of Dutch triplets. BW is affected by zygosity and birth order. MZ triplets were lighter than DZ and TZ triplets, and BW decreased with decreasing birth order in TZ triplets. GA, sex and smoking during pregnancy also had an effect on BW. No effects of ART and alcohol consumption were seen. We did not observe a significant flattening of the BW curve in the last stage of mature triplet gestation. The resemblance for BW was higher in MZ triplets than in DZ triplets after correction for the other risk factors indicating that genetic factors are also of importance. BW discordance in triplets is common since in 21.5% of the trios the difference in BW between the heaviest and lightest triplet was more than 30%.

### Factors affecting triplet BW

The most important factor in triplet BW is GA. As expected, in this sample a higher GA was associated with a higher BW in triplets. The literature describes the occurrence of a flattening of the growth curve during the last weeks of triplet pregnancies. This growth restriction period emerges round a GA of about 36 to 37 weeks [17]. In the NTR sample only 1.5% of triplet pregnancies reached a GA of 37 weeks or more and no flattening of the BW growth curve was seen.

The effects of alcohol consumption and smoking during pregnancy on BW were both examined. No effect of alcohol consumption on triplet BW was seen. We hypothesize that in this sample the absence of an effect was seen because of the low maternal alcohol consumption during pregnancy. In addition, mothers reported less consistent on alcohol consumption than on other variables.

Mothers of triplets who smoked had children who were on average around 100 grams lighter than non-smoking mothers, which is a decline in BW of 4%. This is the effect of smoking after correcting for the GA of the triplet. Studies in singletons report that children of smoking mothers are 119 to 241 grams lighter, which is 4 to 7% lighter than children of non-smoking mothers. The amount of loss in BW is dependent on the quantity the mother smoked during the pregnancy. Within the NTR group of smoking mothers around 70% smoked 0 to 5 cigarettes per day, 15% smoked 5 to 10, and about 15% smoked more than 10 cigarettes per day. In singletons an interaction between alcohol consumption and smoking has also been observed. We did not test for such an effect because only 3% of the triplet mothers both consumed alcohol and smoked.

First born TZ triplets were heavier than the second born TZ triplets, who were heavier than the third born children. No significant birth order effect was seen in the group of MZ and DZ triplets. As the MZ and DZ groups were small, this might reflect a lack of power to detect a difference. In twins, the first born (and heavier) twin, has an heavier placenta and a more optimal (a central instead of peripheral) cord insertion [41]. Possibly triplets higher in birth order are, on average, more optimally positioned with respect to nutrients intake.

### BW discordance and SGA

The prevalence of BW discordance is comparable with a study of Jacobs et al. (2003) [42] and other studies (for a short review see Blickstein et al. (2002) or Blickstein & Kalish (2003) [15,34]). Compared to singletons or twins, triplets are delayed in growth and cognitive development. There has been limited research on the effects of BW discordance, but it seems that triplets who are discordant in BW are at an even higher developmental risk than other triplets [43,44]. One study found that most triplets without BW discordance have caught up with singleton and twin standards on cognitive and executive functions at age 5. In contrast, BW discordant triplets still showed a lower performance on these functions at that age [45]. BW discordance in that study was defined as a difference in BW between the heaviest and lightest triplet of more than 15%. In the current sample, this includes 63.2% of the trios.

We also estimated the resemblance in BW of triplets. The resemblance for BW in MZ triplets was higher than in DZ triplets. Both were higher before correction for GA, sex and smoking than after adjusting for these factors. This suggests that genetic as well as common environmental factors influence birth weight and that GA, sex and smoking are some of the specific common environmental factors.

We observed that, when taking US singleton standards as a reference, 40-50% of the triplets were SGA. Children who are born SGA are at risk for asphyxia and intrauterine mortality [46,47]. As a consequence, children born SGA have to be monitored in neonatal intensive care units (NICU). In the Netherlands triplets are classified SGA based on singleton standards. As a result, at least half of all triplet births have to be born in tertiary referral centers with NICU facilities.

### ART

Population based triplet zygosity distributions have changed over the years. Imaizumi [48] reported that in the Netherlands, the TZ rate increased from 1972-1973 to 1990-1991 and decreased thereafter. Imaizumi concluded that the temporary higher TZ ratio could be attributed to ART. This conclusion is confirmed by the present study in which ART was more common in TZ triplets than in the other zygosity groups.

Studies in singletons report that children born after ART are lower in BW than spontaneously conceived children. In twins the effect of ART is less clear, some studies find an effect, while others do not [49,50]. The reason for lower BW in children born after ART is not completely understood. Investigators suggest that the procedure of ART itself or maternal characteristics (e.g. age, weight, parity) may cause lower BW in children born after ART. In twins, the adverse effects on mean BW associated with ART are possibly balanced by the favorable effect of DZ zygosity as ART increases the prevalence of DZ twinning and DZ twins are heavier than MZ twins. In the present study we found no effect of ART. The effect was neither present in the entire triplet group, nor in the TZ triplet group. Therefore, in present study it can be concluded that the presumed lowering effect of ART on BW was not counterweighted by the higher prevalence of TZ triplets in the ART group.

## Limitations and strengths

The present sample consisted of triplets who were registered at the NTR and whose parents were willing to participate in survey studies. This led to a small positive selection bias. Triplets from families in which all three children are alive 28 days after birth also have more favorable scores on BW and GA. Moreover, parents are possibly more willing to participate in research when the triplets are healthy compared to parents dealing with illnesses of one or more of their children. The NTR sample was more comparable with a selection of Dutch triplets that were still alive 28 after birth, than with a complete group of Dutch triplets including children who died soon after birth. A comparable positive selection bias was found in a study on secular trends in gestational age and birth weights in twins. In this study twins registered at the NTR were compared with a national reference set. The twins registered at the NTR were found to have a higher GA (36.5 (2.4) compared to 35.9 (3.0) weeks) and a higher BW (2498 (550) grams compared to 2459 (615) grams)[51]. As a result of this positive selection bias, percentages of discordant triplets are probably underestimated compared to the total Dutch triplet population. The positive selection bias could also cause an underestimation of the percentage triplets that are classified as SGA. We also do not know whether the effects of the investigated risk factors might be more pronounced in this more vulnerable group.

We investigated whether zygosity influenced triplet BW. MZ triplets are more in competition for nutrients than DZ and TZ triplets. A more direct measure of triplets sharing placenta's and therefore triplet nutrients competition is chorionicity. Information on chorionicity would therefore have been a valuable addition to the information on zygosity. Currently, no reliable information on chorionicity was available.

Parity has been associated with BW but was not included in the analysis of present study, as information on parity was only available for about three-quarter of the mothers. Including parity would therefore have decreased sample size considerably. In an analysis within the reduced sample, there was no significant effect parity on BW.

Some strengths of this study are also noteworthy. The sample is relatively large. We do not know of another study that took so many risk factors into account analyzing their effect on triplet BW. This study is the first to describe triplet zygosity in the Netherlands based on individual zygosity measures instead of population based estimated zygosity distribution. We therefore could confirm the assumption that ART has inflated the Dutch TZ triplet population. Furthermore, this study is the first to investigate the effect of maternal smoking and alcohol consumption during pregnancy on triplet BW. In addition, although our sample is somewhat positively biased when comparing it to all triplets born in the Netherlands, it is a representative sample for the Dutch triplet population that is still alive one month after birth.

## Conclusions

Longitudinal data collection on triplets is scarce. Data collection within the Netherlands Twin Register (NTR) is broad, including an important focus on behavior. The data collection in triplets that we are currently establishing is unique in its kind. With this dataset it is possible to study long term effects of low BW in triplets, both on physiologic and also on behavioral level. This study was limited to a description of the sex and zygosity distribution of the triplets and the effect of a number of BW characteristics. We found an effect of GA, sex, birth order, zygosity and maternal smoking on triplet birth weight, but found no effect of ART and maternal alcohol consumption. The combined effects implied that differences of 364 grams can be observed between MZ girl triplets of smoking mothers compared to TZ boy triplets of non-smoking mothers of the same GA. Furthermore, we found that MZ triplets resembled each other more than DZ triplets, indicating that, in addition to environmental factors, genetic factors contribute to triplet BW.

## List of abbreviations

ART: assisted reproductive techniques; BW: birth weight; DZ: dizygotic; GA: gestational age; MZ: monozygotic; NICU: neonatal intensive care units; NPR: Netherlands Perinatal Registry; NTR: Netherlands Twin Register; TZ: trizygotic.

## Competing interests

The authors declare that they have no competing interests.

## Authors' contributions

DJL: performed the statistical analyses and participated in the design of the study, data acquisition, interpretation of the statistical analyses, and the draft of the manuscript. CMM: participated in the design of the study, interpretation of the statistical analyses, and draft of the manuscript. CEMB: participated in the data acquisition. JMV: participated in the data acquisition. MCH: participated in the interpretation of the statistical analyses. DIB: participated in the design of the study, interpretation of the statistical analyses, and draft of the manuscript. All authors read and approved the final manuscript.

## Pre-publication history

The pre-publication history for this paper can be accessed here:

http://www.biomedcentral.com/1471-2431/11/24/prepub
